# Draft Genome Sequence of Tombunodavirus UC1

**DOI:** 10.1128/genomeA.00655-15

**Published:** 2015-07-02

**Authors:** Alexander L. Greninger, Joseph L. DeRisi

**Affiliations:** a Department of Biochemistry and Biophysics, UCSF, San Francisco, California, USA; b Howard Hughes Medical Institute, UCSF, San Francisco, California, USA

## Abstract

We report here the draft genome sequence of tombunodavirus UC1 assembled from metagenomic sequencing of organisms in San Francisco wastewater. This virus shares hallmarks of members of the *Tombusviridae* and the nodavirus-like *Plasmopara halstedii* and *Sclerophthora macrospora* viruses.

## GENOME ANNOUNCEMENT

*Tombusviridae* and *Nodaviridae* are both families of single-stranded RNA viruses that demonstrate a wide range of host organisms (1, 2). While performing weekly metagenomic sequencing of organisms in San Francisco wastewater, we assembled a contig of 4,244 nucleotides that by BLASTx aligned 35% at the amino acid level to the RNA-dependent RNA polymerase of the tombusviruses olive latent virus 1 and tobacco necrosis virus and 57% at the amino acid level to the coat protein of the nodavirus-like *Plasmopara halstedii* virus A (3). In addition to these alignments, the first portion of the contig aligned to pfam08500, representing the p33 replication accessory protein from the *Tombusviridae* family (4). The genome size of 4,244 nucleotides is consistent with that of other members of the *Tombusviridae* family, which range from 3.6 kb to 4.8 kb.

We have given the name tombunodavirus UC1 to this virus due to its sequence identity to members of both the nodavirus and tombuvirus families, although it appears the viral genome is monopartite, like a tombusvirus. The contig contains three separate open reading frames (ORFs) in the standard genetic code of 888, 1,482, and 1,353 nucleotides with an overlap of 29 nucleotides, while in a more permissive ciliate genetic code, as the dominant host organism present in the sample, it contains two ORFs of 2,493 and 1,626 nucleotides, representing the unique origins of the two ORFs, which overlap by 164 nucleotides. Members of the *Tombusviridae* family infect plants, while members of the nodavirus family infect a broad array of organisms, including fish and insects. The host organism of tombunodavirus UC1 is currently unknown.

This viral genome was recovered from a wastewater sample from 25 January 2010 that was taken after a large rainstorm left >5 in. of rain over the preceding week. This same sample also contained novel ciliate and marine RNA viruses and phages (5–7). Sample processing was performed on 1 liter of wastewater that was concentrated to <5 ml, with particles between the size of 0.22 µm and 300 kDa using Millipore Pellicon XL 300-kDa filters and 0.22-µm spin columns. Nucleic acid was extracted using the Zymo Viral DNA/RNA kit, and half of the recovered nucleic acid was treated with DNase. The contig was discovered and assembled using PRICE version 1.0 and SURPI version 1.0 from a total of 15,719,690 paired-end 65-bp reads sequenced on an Illumina GAIIx split between these DNAsed and untreated nucleic acid preparations (8, 9). The average coverage of the tombunodavirus contig was 5,089× with coverage 2.4× higher in the DNAsed library than that in the untreated library, consistent with an RNA genome.

### Nucleotide sequence accession number.

The GenBank accession number for tombunodavirus UC1 is KF510030.
